# Supplementary material for: Ex Vivo Drug Sensitivity Correlates with Clinical Response and Supports Personalized Therapy in Pediatric AML
Source: Cancers (Basel). 2022 Dec 18;14(24):6240. doi: 10.3390/cancers14246240 (PMC9777060; doi:10.3390/cancers14246240)
Supplement: Supplementary file 1 [file cancers-14-06240-s001.zip › cancers-2039156-supplementary.pdf]

Table S1. Dose conditions included in the ex vivo drug sensitivity platform. Patient cells were screened against up to 78 dose conditions that include 31 drugs and 47 drug combinations.

**Dose conditions tested in the ex vivo drug sensitivity platform**
5-azacytidine: 1000 nM
5-azacytidine: 1000 nM; Atovaquone: 30000 nM
5-azacytidine: 1000 nM; Atovaquone: 30000 nM; Venetoclax: 225 nM
5-azacytidine: 1000 nM; Atovaquone: 30000 nM; Venetoclax: 25 nM
5-azacytidine: 1000 nM; Venetoclax: 225 nM
5-azacytidine: 1000 nM; Venetoclax: 25 nM
Arsenic Trioxide: 500 nM
Arsenic Trioxide: 500 nM; Atovaquone: 30000 nM
Arsenic Trioxide: 500 nM; Atovaquone: 30000 nM; Tretinoin: 50 nM
Arsenic Trioxide: 500 nM; Tretinoin: 50 nM
Atovaquone: 100 nM
Atovaquone: 1000 nM
Atovaquone: 10000 nM
Atovaquone: 20000 nM
Atovaquone: 30000 nM
Atovaquone: 30000 nM; Bortezomib: 11 nM; Panobinostat: 5 nM
Atovaquone: 30000 nM; Calcitriol: 1 nM
Atovaquone: 30000 nM; Calcitriol: 1 nM; Dexamethasone: 200 nM
Atovaquone: 30000 nM; Crenolanib: 20 nM
Atovaquone: 30000 nM; Cytarabine: 300 nM
Atovaquone: 30000 nM; Cytarabine: 300 nM; Daunorubicin Hydrochloride: 70 nM
Atovaquone: 30000 nM; Cytarabine: 300 nM; Daunorubicin Hydrochloride: 70 nM; Etoposide: 300 nM
Atovaquone: 30000 nM; Cytarabine: 300 nM; Etoposide: 300 nM
Atovaquone: 30000 nM; Cytarabine: 300 nM; Etoposide: 300 nM; Sorafenib: 40 nM
Atovaquone: 30000 nM; Daunorubicin Hydrochloride: 70 nM
Atovaquone: 30000 nM; Decitabine: 1000 nM
Atovaquone: 30000 nM; Decitabine: 1000 nM; Venetoclax: 225 nM
Atovaquone: 30000 nM; Decitabine: 1000 nM; Venetoclax: 25 nM
Atovaquone: 30000 nM; Dexamethasone: 200 nM
Atovaquone: 30000 nM; Enasidenib: 500 nM
Atovaquone: 30000 nM; Etoposide: 300 nM
Atovaquone: 30000 nM; Gilteritinib: 3 nM
Atovaquone: 30000 nM; Ivosidenib: 600 nM
Atovaquone: 30000 nM; Midostaurin: 100 nM
Atovaquone: 30000 nM; Mivebresib: 20 nM
Atovaquone: 30000 nM; Panobinostat: 5 nM
Atovaquone: 30000 nM; Quizartinib: 20 nM
Atovaquone: 30000 nM; Rucaparib: 1000 nM
Atovaquone: 30000 nM; Sorafenib: 40 nM
Atovaquone: 30000 nM; Sunitinib: 10 nM

Atovaquone: 30000 nM; Trametinib: 10 nM
Atovaquone: 30000 nM; Tretinoin: 50 nM
Atovaquone: 30000 nM; Venetoclax: 225 nM
Atovaquone: 30000 nM; Venetoclax: 25 nM
Atovaquone: 30000 nM; Vorinostat: 300 nM
Bortezomib: 11 nM
Bortezomib: 11 nM; Panobinostat: 5 nM
Calcitriol: 1 nM
Calcitriol: 1 nM; Dexamethasone: 200 nM
Crenolanib: 20 nM
Cytarabine: 300 nM
Cytarabine: 300 nM; Daunorubicin Hydrochloride: 70 nM
Cytarabine: 300 nM; Daunorubicin Hydrochloride: 70 nM; Etoposide: 300 nM
Cytarabine: 300 nM; Etoposide: 300 nM
Cytarabine: 300 nM; Etoposide: 300 nM; Sorafenib: 40 nM
Daunorubicin Hydrochloride: 70 nM
Daunorubicin Hydrochloride: 70 nM; Etoposide: 300 nM
Decitabine: 1000 nM
Decitabine: 1000 nM; Venetoclax: 225 nM
Decitabine: 1000 nM; Venetoclax: 25 nM
Dexamethasone: 200 nM
Enasidenib: 500 nM
Etoposide: 300 nM
Gilteritinib: 3 nM
Ivosidenib: 600 nM
Midostaurin: 100 nM
Mivebresib: 20 nM
Panobinostat: 100 nM
Panobinostat: 5 nM
Quizartinib: 20 nM
Rucaparib: 1000 nM
Sorafenib: 40 nM
Sunitinib: 10 nM
Trametinib: 10 nM
Tretinoin: 50 nM
Venetoclax: 225 nM
Venetoclax: 25 nM
Vorinostat: 300 nM

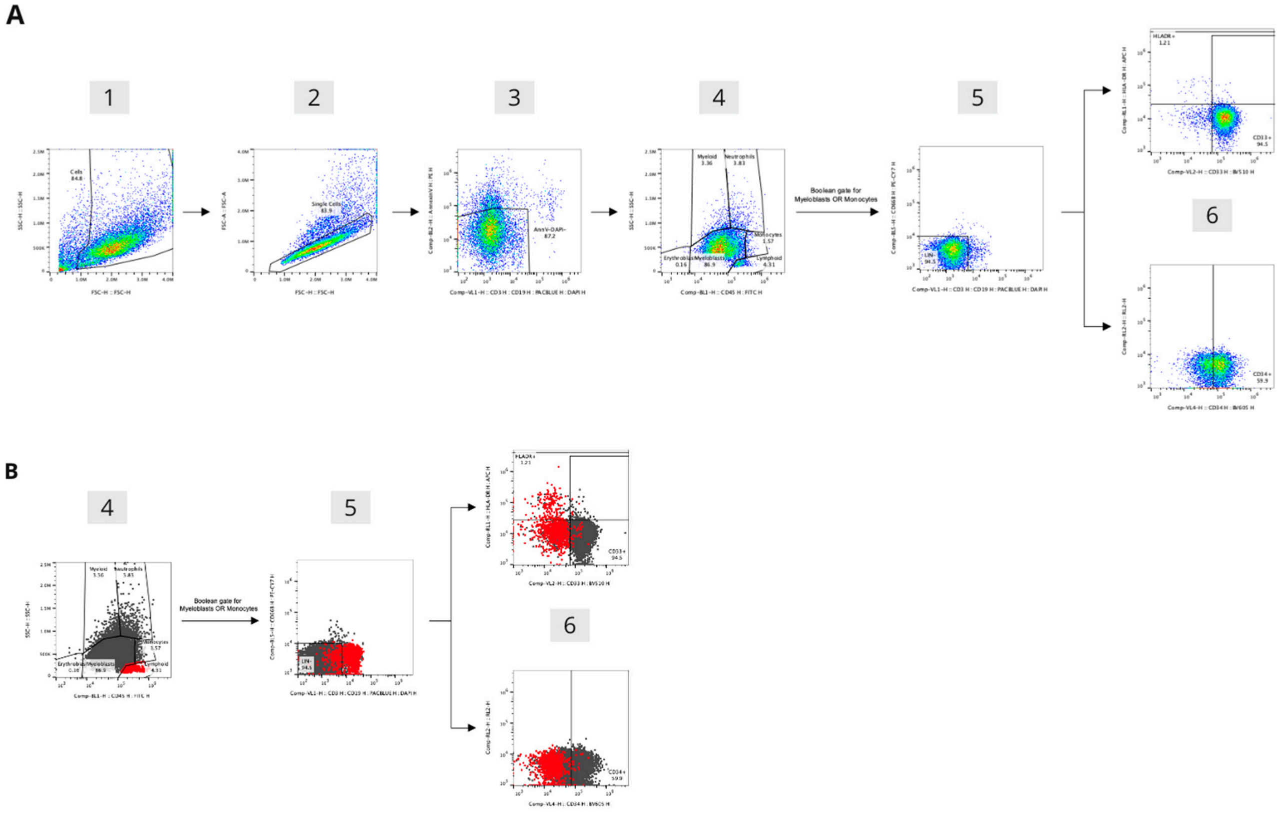

Figure S1. Gating strategy to identify total blasts in patient samples. (**A**) Gate 1: FSC x SSC was used to identify "Cells". Gate 2: "Single Cells" were defined as having a 1:1 FSC-H x FSC-A. Gate 3: Live cells were defined as "AnnV-DAPI-" in the lower left quadrant. Gate 4: Leukocyte populations as labeled were defined by CD45 x SSC. A Boolean gate was created that included both "Myeloblasts" and "Monocytes". From this Boolean gate (Myeloblasts + Monocytes), Gate 5: "LIN-" cells were defined as being negative for CD66b, CD3 and CD19. Gate 6: "Total Blasts" were defined using a Boolean gate to include LIN- cells that express CD34, HLA-DR or CD33. (**B**) Large dot overlay of Lymphoid cells in red for gates 4-6 from (A).

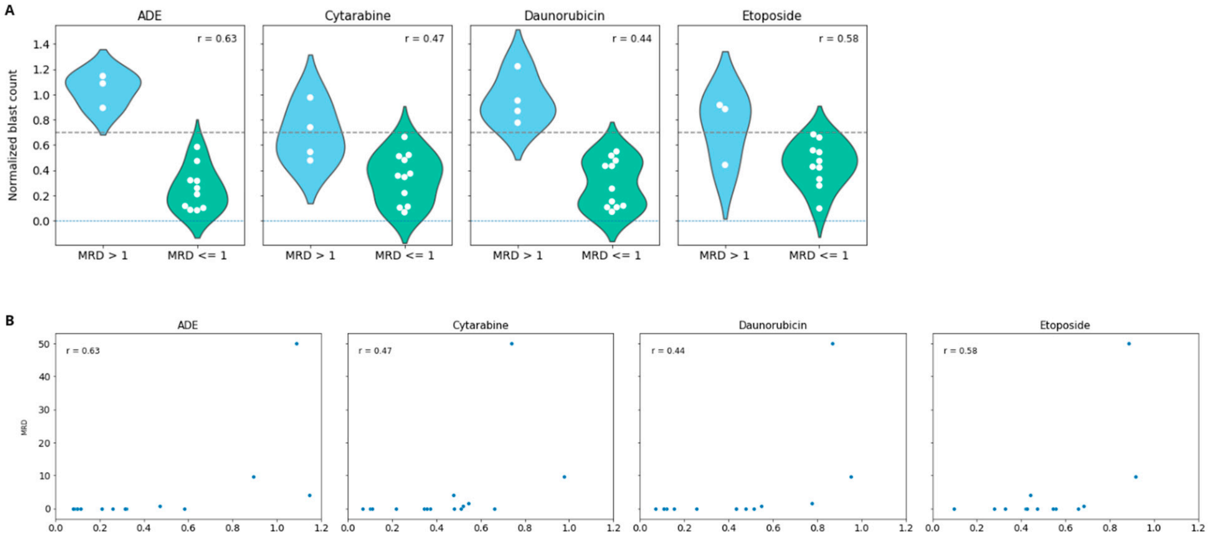

Figure S2. Ex vivo drug sensitivity for ADE shows the highest correlation with MRD compared to single agents. Diagnostic samples from 13 de novo pediatric AML patients that received ADE were screened for sensitivity ex vivo to ADE (cytarabine, daunorubicin and etoposide) in single agent and combination settings. (**A**) Patient samples were binarized into two groups by MRD at end of induction: MRD > 1 (blue; n=10) and MRD <= 1 (green; n=3). (**B**) Scatterplot of normalized blast counts compared to MRD reported for each patient sample. Correlation between MRD and ex vivo drug sensitivity for each single agent and the ADE combination is shown.

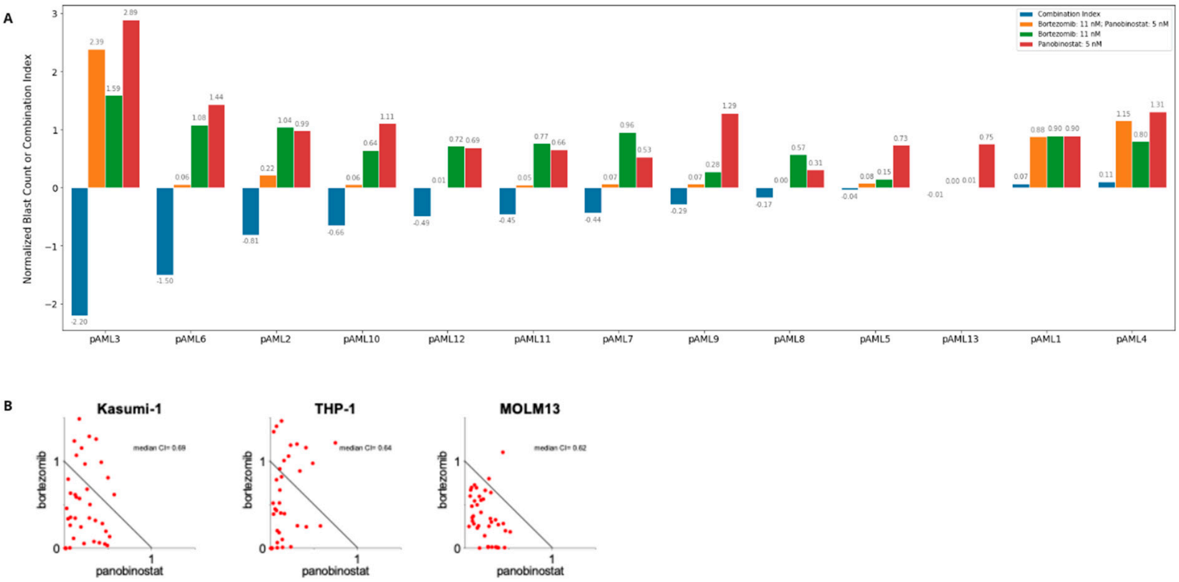

Figure S3. Bortezomib and panobinostat show combination activity in patient samples and AML cell lines. (**A**) Diagnostic samples from 13 de novo pediatric AML patients were screened for sensitivity to bortezomib and panobinostant in single agent and combination settings. Normalized blast counts in response to bortezomib (green), panobinostat (red), and bortezomib/panobinostat (orange) are labeled. Combination Index (blue bars) was calculated as the average observed effect minus the average expected effect, where the average expected effect is the product of the normalized total blast values. (**B**) Drug combination assay for bortezomib and panobinostat was performed in luciferase transduced Kasumi-1, THP-1, and MOLM13 cell lines. Cells were plated at 10e4 per well in 96-well plates with medium only, single drugs at increasing concentrations, and panobinostat+bortezomib combinations (panobinostat 0.38-680.09nM, bortezomib 1.48-26.43nM). After 48 hours, viability was determined by luminescence (Luminoskan ascent, Thermo electron). Background luminescence in medium-only wells was subtracted from all values and means of 4 replicates were used for analysis. All values were normalized to cells + vehicle wells, which were set to 0 to quantify percent affected. Calcusyn was used to calculate the median combination index (CI). A panobinostat+bortezomib combination was considered synergistic if CI<0.8, additive if CI>0.8 and <1.2, or antagonistic if CI>1.2. Three independent experiments were done for each cell line. Representative normalized isobolograms are shown.
